# Loss of fatty acid synthase inhibits the “HER2-PI3K/Akt axis” activity and malignant phenotype of Caco-2 cells

**DOI:** 10.1186/1476-511X-12-83

**Published:** 2013-06-01

**Authors:** Nan Li, Heng Lu, Chunyan Chen, Xiaodong Bu, Peilin Huang

**Affiliations:** 1 Department of Gastroenterology and Hepatology, Jinling Hospital, Nanjing University School of Medicine, Nanjing, 210002, Peoples R China; 2 Department of Pathology, Southeast University School of Medicine, Nanjing, 210009, People's Republic of China

**Keywords:** FASN, “HER2-PI3K/Akt axis”, Malignant phenotype, Caco-2 cells

## Abstract

**Background:**

Fatty acid synthase (FASN) is frequently activated and overexpressed in human cancers, and plays a crucial role in the carcinogenesis of various cancers. In this study, our aims were to explore the role of FASN in regulating the “HER2-PI3K/Akt axis” activity and malignant phenotype of colorectal cancer.

**Methods:**

Caco-2 cells with a high expression of both HER2 and FASN were selected for functional characterization. Caco-2 cells were transfected with either the FASN specific RNAi plasmid or the negative control RNAi plasmid, followed by the RT-qPCR and western blot to examine the expression of FASN, HER2, PI3K and Akt. The MTT and colony formation assays were used to assess the proliferation potential. The migration was investigated by the transwell, and the apoptosis and cell cycle were assayed by the flow cytometry.

**Results:**

Notably, the expression of FASN, HER2, PI3K and Akt were downregulated upon a silence of FASN. The proliferation was decreased after a downregulation of FASN, which was consistent with an increased apoptosis rate. The migration was also impaired in FASN-silenced cells.

**Conclusion:**

A downregulation of FASN effectively inhibits the activity of “HER2-PI3K/Akt axis” and alters the malignant phenotype in colorectal cancer cells.

## Background

Fatty acid synthase (FASN) is a homodimeric multienzymatic protein which can be divided into seven functional domains that are assembled into two homodimers [1]. Through a series of reactions, FASN synthesizes the long chain fatty acids (LCFA) using acetyl-CoA and malonyl-CoA as substrates and NADPH as an electron donor, respectively [2]. FASN is expressed by the lipogenic tissues, hormone-sensitive cells and proliferating fetal cells [3]. In normal cells, FASN expression maintains at a low level and its regulation is a complex process highly relying on the nutritional status and hormonal profile [4,5]. However, in cancer cells and pre-neoplastic lesions, FASN expression has been frequently found to be upregulated. An increased FASN expression is associated with the cancer progression, higher risk of recurrence and shorter survival in many types of cancers [6-8].

An increased FASN expression renders cancer cells no longer responsive to the nutritional cue [9]. Though there are many potential causes for its upregulation, the transcriptional regulation of FASN expression has been considered to be the major cause for the increased FASN expression in cancer cells [10-12]. It has been shown that the growth factors, hormones and activation of their receptors increased FASN transcription in cancer cells. For example, epidermal growth factor (EGF) can stimulate FASN expression through the EGF receptor ERBB1 and ERBB2 (HER2; Her-2/neu) [10,11]. Furthermore, the effect of growth factors, hormones and their receptors on FASN expression involves a complicated downstream signaling and crosstalk between multiple signal transduction pathways. A well-studied major pathway that is possibly involved in regulating FASN expression is the PI3K/Akt pathway. Previously, many studies have demonstrated the link between the PI3K/Akt activity and FASN expression [13,14].

A transcriptome analysis of HER2 in breast cancer cells has revealed a molecular connection between FASN and HER2 through the PI3K/Akt pathway [10]. In this study, the authors used DNA microarray to compare and identify genes induced by HER2 in mammary epithelial cell line with ectopic HER2 overexpression and breast cancer cell lines derived from patients with different level of HER2 expression. They found that HER2 overexpression activated FASN promoter and transcription as well as increased protein production and activity, while inhibitors of HER2, Herceptin and CI-1003, attenuated the effect of HER2 on FASN expression. PI3K activity was thought to be the mediator of the HER2 control on FASN expression because LY294002, a known PI3K inhibitor, abrogated HER2 induced FASN protein production in the HER2 overexpressing normal mammary epithelial and breast cancer cells. Thus, the transcription of FASN gene may be induced by HER2 via the PI3K/Akt pathway. Conversely, FASN dependent regulation of HER2 expression has also been reported [15].

Hence, in this study, we analyzed the potential link between FASN and the activity of “HER2-PI3K/Akt axis” in colorectal cancer cells. And the influence of FASN on proliferation and migration of colorectal cancer cells was also explored.

## Materials and methods

### Cell culture and selection

Four human colorectal cancer cells of Caco-2, HT-29, LoVo and LS174T were used in this study. All cells were purchased from Shanghai Cell Biology Institute of Chinese Academy of Sciences (Shanghai, China). HT-29, LoVo and LS174T cells were cultured in RPMI-1640 medium supplemented with 10% fetal bovine serum (Invitrogen, USA). Caco-2 cells were cultured in minimum essential medium (MEM) supplemented with 10% fetal bovine serum (Invitrogen, USA). All cells were incubated at 37°C in a humidified atmosphere supplemented with 5% CO_2_. HER2 and FASN mRNA expression of four cells were detected by the RT-qPCR. A negative control RNAi plasmid with the scrambled sequences (marked as MR-Neg) (Table 1) was synthesized according to the manufacturer’s instructions (Invitrogen, USA), and four cells were transiently transfected with the MR-Neg to detect the transfection efficiency. HER2 and FASN mRNA expression and the transfection efficiency of four cells were integrated for selecting target cells.

**Table 1:** The oligo sequences of four FASN specific RNAi plasmids and the negative control RNAi plasmid

**Plasmid**		**Oligo sequence (5′-3′)**
**MR-FASN-1**	Forward	TGCTGAACTCCTGCAAGTTCTCCGACGTTTTGGCCACTGACTGACGTCGGAGATTGCAGGAGTT
	Reverse	CCTGAACTCCTGCAATCTCCGACGTCAGTCAGTGGCCAAAACGTCGGAGAACTTGCAGGAGTTC
**MR-FASN-2**	Forward	TGCTGAGAAGAGGCTCTCCCACGCGTGTTTTGGCCACTGACTGACACGCGTGGGAGCCTCTTCT
	Reverse	CCTGAGAAGAGGCTCCCACGCGTGTCAGTCAGTGGCCAAAACACGCGTGGGAGAGCCTCTTCTC
**MR-FASN-3**	Forward	TGCTGATACCTAGCAGGCTGTCCTGGGTTTTGGCCACTGACTGACCCAGGACACTGCTAGGTAT
	Reverse	CCTGATACCTAGCAGTGTCCTGGGTCAGTCAGTGGCCAAAACCCAGGACAGCCTGCTAGGTATC
**MR-FASN-4**	Forward	TGCTGTAGCCAAGCACCTCACGCTGGGTTTTGGCCACTGACTGACCCAGCGTGGTGCTTGGCTA
	Reverse	CCTGTAGCCAAGCACCACGCTGGGTCAGTCAGTGGCCAAAACCCAGCGTGAGGTGCTTGGCTAC
**MR-Neg**	Forward	tgctgAAATGTACTGCGCGTGGAGACGTTTTGGCCACTGACTGACGTCTCCACGCAGTACATTT
	Reverse	cctgAAATGTACTGCGTGGAGACGTCAGTCAGTGGCCAAAACGTCTCCACGCGCAGTACATTTc

### Plasmid construction and stable transfectional cells establishment

Knockdown of FASN was achieved with an RNA interference approach using microRNA to obtain the stable clones. Four different FASN specific RNAi plasmids (marked as MR-FASN-1—MR-FASN-4) were synthesized according to the manufacturer’s instructions (Invitrogen, USA) (Table 1), and Caco-2 cells were transiently transfected with them, respectively. FASN mRNA expression was detected by the RT-qPCR to validate knockdown effect and choose the most effective FASN specific RNAi plasmid. Then Caco-2 cells were respectively transfected with the most effective FASN specific RNAi plasmid (MR-FASN-2) and the negative control RNAi plasmid (MR-Neg) using Lipofectamine 2000 according to the Invitrogen technical bulletin. Blasticidin (7 μg/ml) was used to select for the stable clones.

### Real-time quantitative polymerase chain reaction

Total RNA was isolated with the TRIzol method (Invitrogen, USA). cDNA was synthesized by a reverse transcription system kit (Invitrogen, USA) according to the manufacturer’s instruction. Gene mRNA expression was verified using a fluorescence quantitative PCR system (BioRad, USA). Glyceraldehyde-3-phosphate dehydrogenase (GAPDH) was used as an internal standard. The gene primer sequences were listed in Table 2. The cycling conditions were as follows: initial denaturation at 95°C for 2 min, followed by 40 amplification cycles of 95°C for 10 s, 60°C for 30 s, and 70°C for 45 s. Every real-time PCR assay contained 1.2 μl cDNA template, 0.5 μl SYBR green, and 0.5 μl of every forward and reverse primer in a 25 μl reaction mixture. Relative gene mRNA expression was analyzed using the 2^-ΔΔCT^ method [16].

**Table 2:** The gene primer sequences

**Gene**		**Primer sequence (5′-3′)**
**HER2**	Forward	CACTGCCAACCGGCCAGAGG
	Reverse	GACACTCAGGGTGGCACGGC
**PI3K**	Forward	TGTAGTGGTGGACGGCGAAGTA
	Reverse	GGGAGGTGTGTTGGTAATGTAGCA
**Akt**	Forward	GAGAGGAGCGCGTGAGCGTC
	Reverse	TCATCAGCTGGCACTGCGCC
**FASN**	Forward	CGACAGCACCAGCTTCGCCA
	Reverse	CACGCTGGCCTGCAGCTTCT
**GAPDH**	Forward	GAAGGTCGGAGTCAACGGATT
	Reverse	CGCTCCTGGAAGATGGTGAT

### Western blot analysis

Equal amounts of protein (100 μg per lane) were subjected to a 4%-12% NuPAGE Novex Bis-Tris Mini Gels (Invitrogen, USA) and the separated proteins were transferred onto the Immobilon P PVDF membrane (Invitrogen, USA). The membranes were blotted using primary antibodies directed against human FASN (1:500, Cell Signaling Technology, USA), HER2, PI3K, Akt and phosphAkt (1:500, Signalway Antibody, USA). After incubation with the appropriate antirabbit or antimouse horseradish peroxidase-conjugated secondary antibody (1:10000, Santa Cruz, CA), immunoreactive bands were visualized by the chemiluminescence dissolvent and exposured to the X-ray film. GAPDH (1:10000, Santa Cruz, CA) protein expression was used as a normalization control for protein loading.

### Cell proliferation assay

Cells (6 × 10^3^/200 μl/well) were seeded in 96-well plates. Viable proliferating cells were detected by the 3-(4,-dimethy-lthiazol-2-yl)-2,-diphenyl-tetrazoliumbromide (MTT) assay at various time period (24, 48, 72, 96, and 120 h), using six wells per time period. Cell viability was expressed as optical density (OD), which was detected by an enzyme-linked immunoabsorbent assay reader (Thermo, MK3, USA) at 492 nm wavelength.

### Colony formation assay

Cells (6 × 10^2^/2 ml/well) were seeded in 6-well plates, and cultured for 2 weeks to form colonies. The formed colonies were stained with Giemsa, and the colonies containing more than 50 cells were counted under an inverted microscope.

### Cell migration assay

Cell migration was measured in 24-well plates by the transwell assay using a chamber containing the polyethylene terephthalate filter membrane with 8-μm pores (Corning, USA). Cells (6 × 10^4^/200 μl/chamber) were seeded in the upper chamber with MEM, and 500 μl MEM supplemented with 10% fetal bovine serum was filled in the lower well as a chemoattractant. After incubation for 24 h, the chambers were stained with hematoxylin-eosin (HE). The migrated cells were counted from five randomly selected fields under an inverted microscope.

### Cell apoptosis analysis

Cells (5 × 10^5^) were harvested, washed with PBS and resuspended in Binding Buffer (Kaijibio, China), followed by mixing with Annexin V-FITC and Propidium iodide (Kaijibio, China). Cells were analyzed by a Becton-Dickinson FACSCalibur flow-cytometer provided with the CellQuest software (Braintree, MA).

### Cell cycle analysis

Cells (1 × 10^6^) were harvested, washed with PBS and fixed in 70% ethanol. The fixed cells were washed with PBS and resuspended in RNase A (Kaijibio, China), followed by incubation at 37°C for 30 min. Cells were stained with PI solution (Kaijibio, China) and analyzed by a Becton-Dickinson FACSCalibur flow-cytometer provided with the CellQuest software (Braintree, MA).

### Statistical analysis

Statistical comparisons were performed by SAS software, version 9.1 (SAS Institute, Cary, NC). Values are presented as means ± standard error of mean (SEM). The statistical significance of differences was determined by One-way ANOVA with a post-hoc test (Student-Newman-Keuls test). Values of *P* < 0.05 were considered to be statistically significant.

## Results

### Cell selection

**Figure 1:**
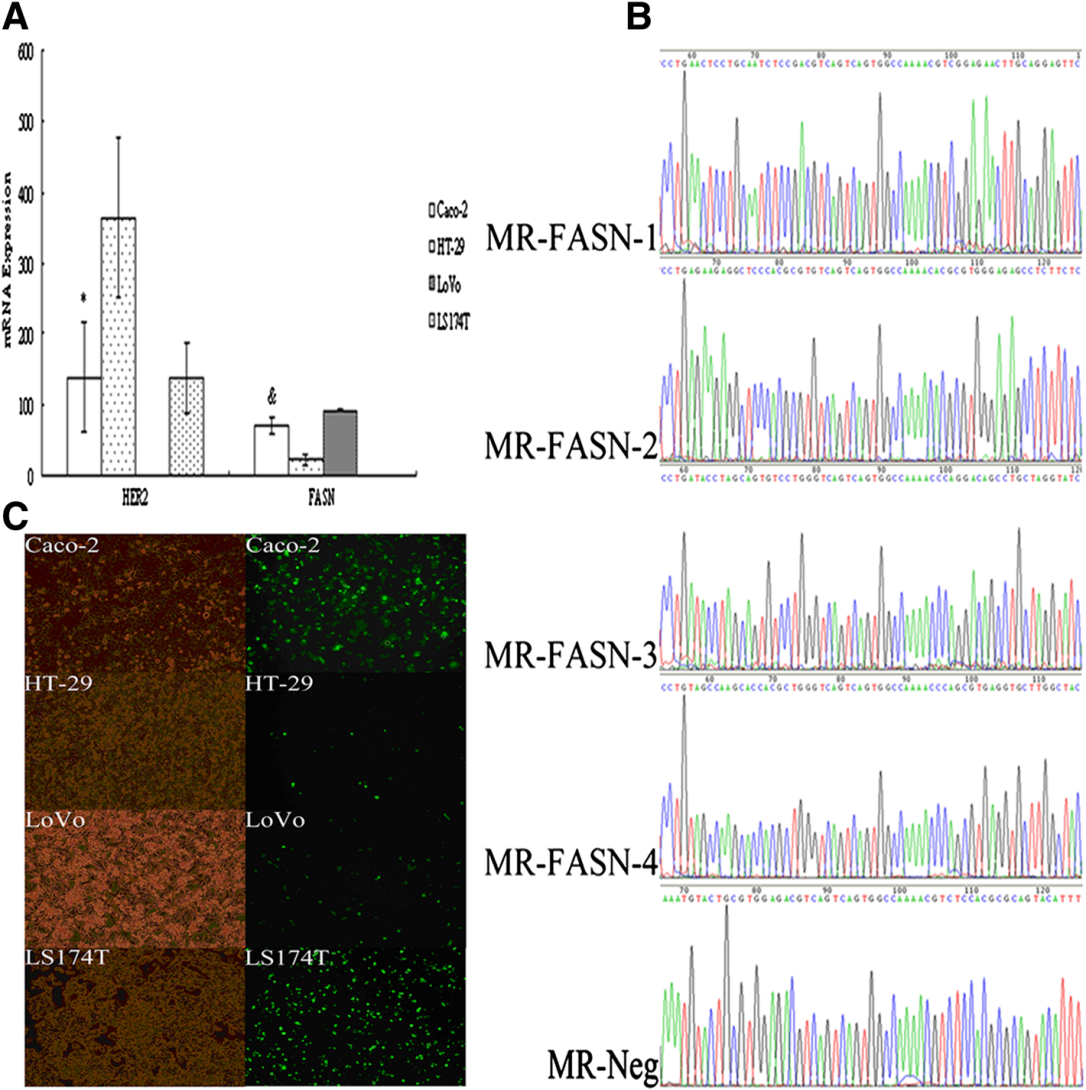
**Cell selection.** (**A**) HER2 and FASN mRNA expression of Caco-2, HT-29, LoVo and LS174T cells. * Compared with HT-29 and LoVo cells, *P* < 0.05; Compared with LS174T cells, *P* > 0.05. ^&^ Compared with HT-29, LoVo and LS174T cells, *P* < 0.05. (**B**) The sequencing of four different FASN specific RNAi plasmids and the negative control RNAi plasmid. (**C**) The white light and fluorescent photographs of Caco-2, HT-29, LoVo and LS174T cells transiently transfected with the negative control RNAi plasmid for 24 h (×100 magnification).

HER2 mRNA expression of Caco-2, HT-29, LoVo and LS174T cells were respectively 138.46 ± 77.6, 365.4 ± 113.48, 1.00 and 137.19 ± 51.32. FASN mRNA expression of them were respectively 70.52 ± 11.53, 22.21 ± 6.41, 92.63 ± 0.98 and 1.00 (Figure 1A). After transiently transfected with the MR-Neg for 24 h (Figure 1B), the transfection efficiency of four cells was investigated by an inverted fluorescence microscope (Figure 1C). Caco-2 cells were the only cell line tested that expressed reasonable levels of both HER2 and FASN and had good plasmid uptake. Hence, Caco-2 cells were selected as the target cells to perform further experiments.

### Plasmid selection and stable transfectional cells establishment

**Figure 2:**
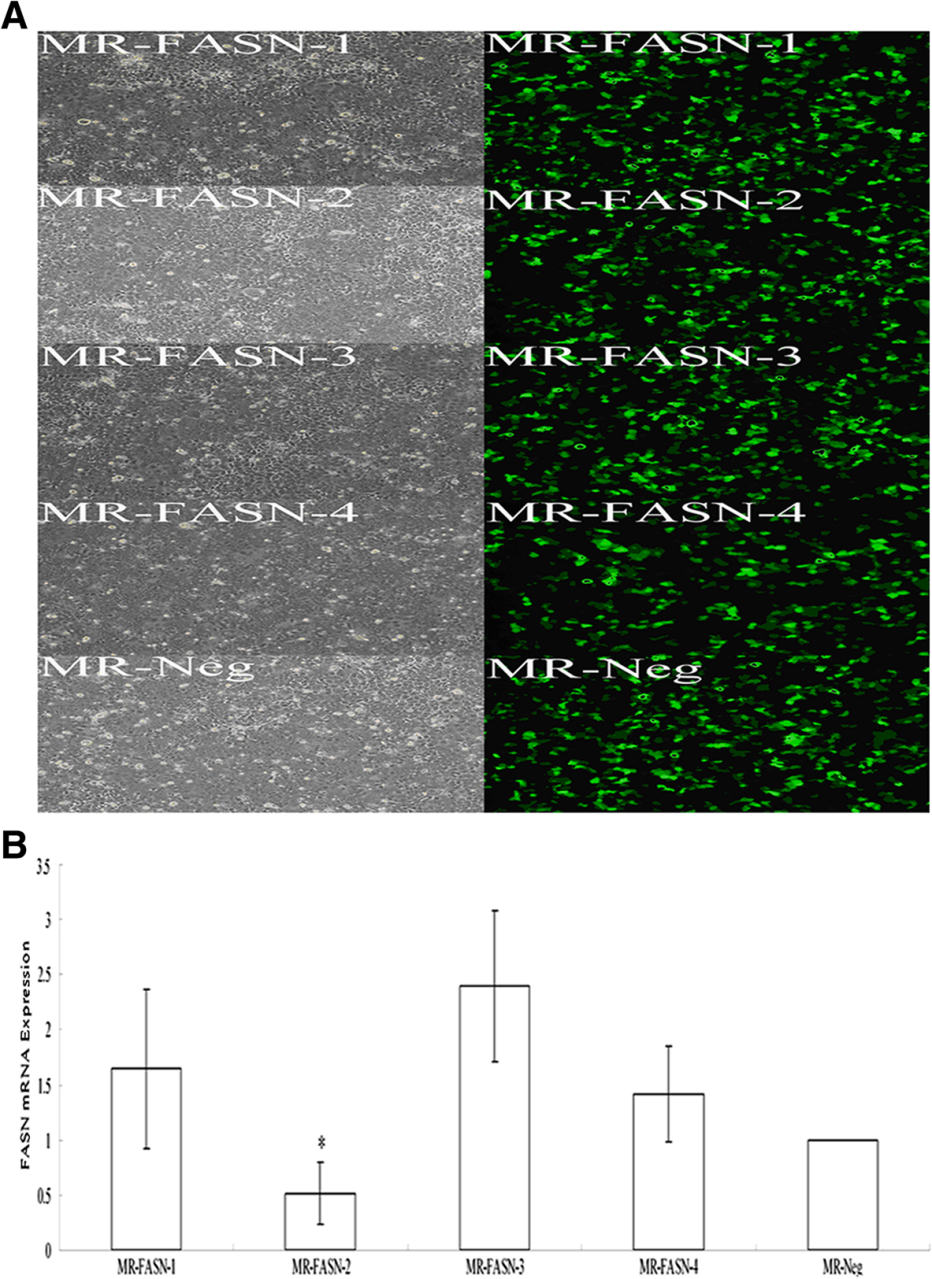
**Plasmid selection.** (**A**) The white light and fluorescent photographs of transfection efficiency of Caco-2 cells transiently transfected with four different FASN specific RNAi plasmids and the negative control RNAi plasmid for 24 h (×100 magnification). (**B**) FASN mRNA expression of Caco-2 cells transiently transfected with four different FASN specific RNAi plasmids and the negative control RNAi plasmid for 24 h. * Compared with other groups, *P* < 0.05.

Upon the transient transfection of four different FASN specific RNAi plasmids for 24 h (Figure 2A), the FASN mRNA expression of Caco-2 cells was 1.64 ± 0.72, 0.52 ± 0.28, 2.4 ± 0.68 and 1.41 ± 0.43. Compared with other groups, FASN mRNA expression of Caco-2 cells transfected with the MR-FASN-2 was the lowest (*P* < 0.05) (Figure 2B). Therefore, Caco-2 cells were transfected with the most effective FASN specific RNAi plasmid (MR-FASN-2) and the negative control RNAi plasmid (MR-Neg), and blasticidin (7 μg/ml) was used to select for stable clones.

### Inhibition of FASN by RNA interference suppressed the activity of “HER2-PI3K/Akt axis” in Caco-2 cells

**Figure 3:**
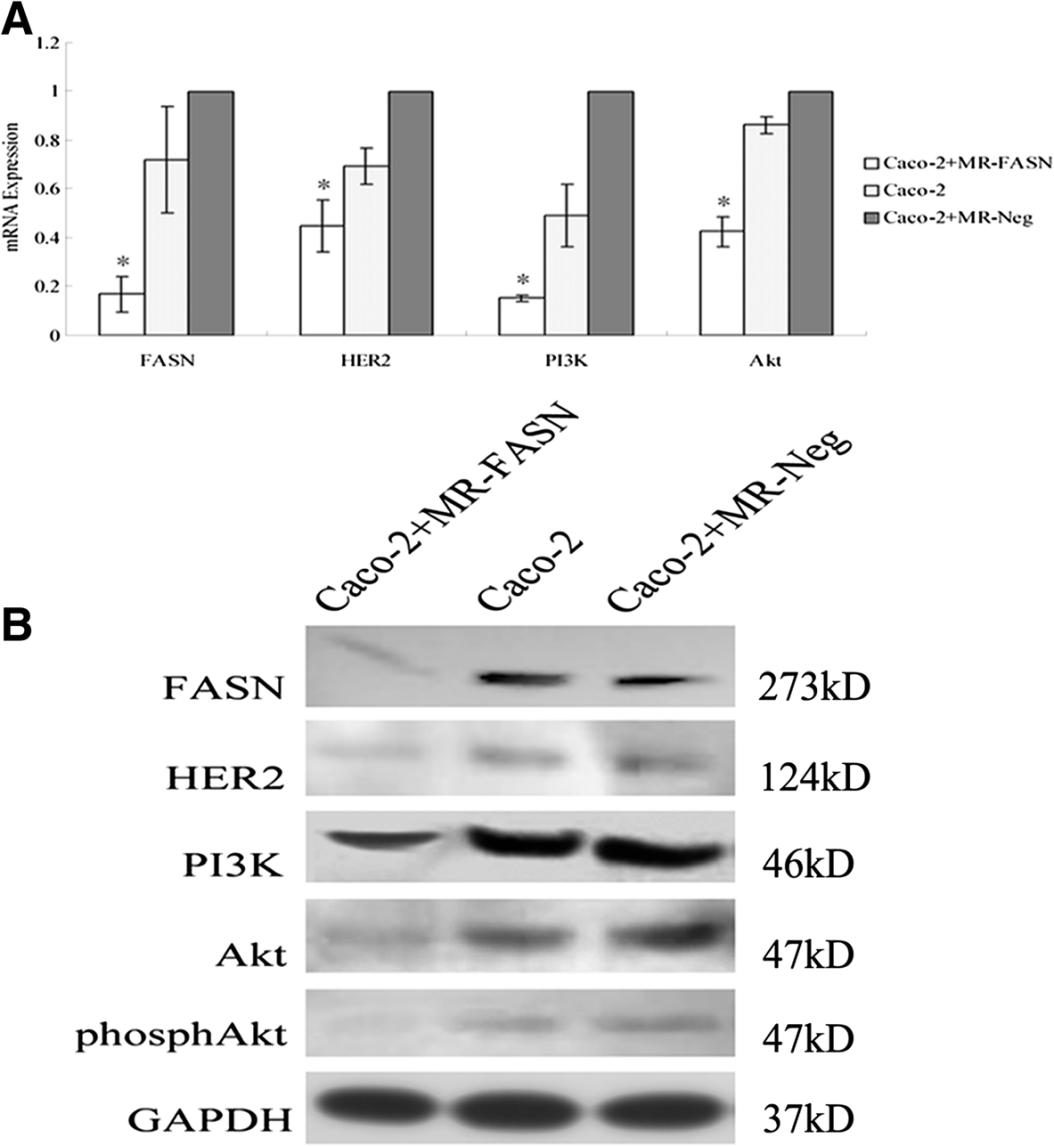
**FASN, HER2, PI3K and Akt expression of Caco-2 cells by RNA interference.** (**A**) FASN, HER2, PI3K and Akt mRNA expression. * Compared with two control groups, *P* < 0.05. (**B**) FASN, HER2, PI3K, Akt and phosphAkt protein expression.

FASN mRNA expression of experimental group was 0.17 ± 0.07, significantly lower than the control groups (0.72 ± 0.22 and 1.00) (*P* < 0.05) (Figure 3A). FASN protein expression was also decreased, compared to two control groups (Figure 3B). Interestingly, after inhibiting FASN expression, HER2, PI3K and Akt mRNA expression of FASN-RNAi group were 0.45 ± 0.11, 0.15 ± 0.01 and 0.42 ± 0.06, all significantly lower than the control groups (0.69 ± 0.07 and 1.00, 0.49 ± 0.13 and 1.00, 0.86 ± 0.03 and 1.00) (*P* < 0.05) (Figure 3A). Correspondingly, HER2, PI3K and Akt protein expression were also dramatically declined (Figure 3B). In addition, western blot analysis showed a significant decrease in phosphAkt, as well (Figure 3B).

### Inhibition of FASN blocked proliferation and migration of Caco-2 cells

**Figure 4:**
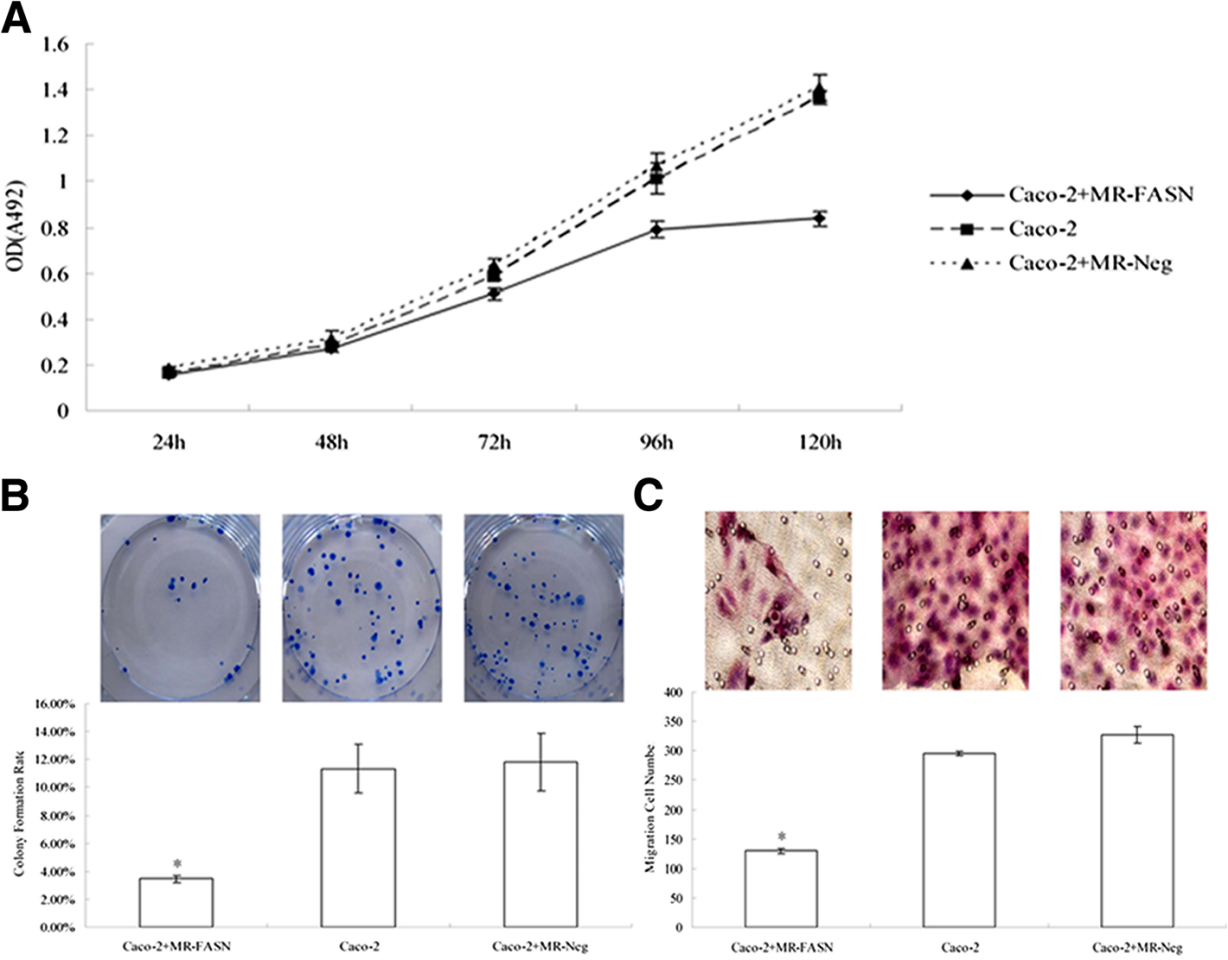
**The proliferation and migration of Caco-2 cells by RNA interference.** (**A**) The proliferation curve. Compared with two control groups, *P* < 0.05. (**B**) The colony formation Rate. * Compared with two control groups, *P* < 0.05. (**C**) The migration cell number. * Compared with two control groups, *P* < 0.05 (HE, ×200 magnification).

To examine the possible involvement of FASN in cancer progression, the proliferation and migration of Caco-2 cells after silencing FASN were assessed using the MTT, colony formation and transwell assays. As shown in Figure 4A, the OD values measured at 5 days showed that the proliferation of experimental group was significantly lower than two control groups (*P* < 0.05). The colony formation rate of FASN-silencing group was 3.45% ± 0.003, and was obviously declined, compared with the blank control group (11.33 % ± 0.02) and negative control group (11.78% ± 0.02) (*P* < 0.05) (Figure 4B). In the transwell assay, FASN silenced cells migrated less efficiently than control cells (*P* < 0.05) (Figure 4C). The number of migrating FASN silenced Caco-2 cells was 129 ± 4.36, compared with 295.33 ± 4.04 in the blank control group and 327 ± 14.53 in the negative control group.

### RNA interference induced apoptosis of Caco-2 cells

**Figure 5:**
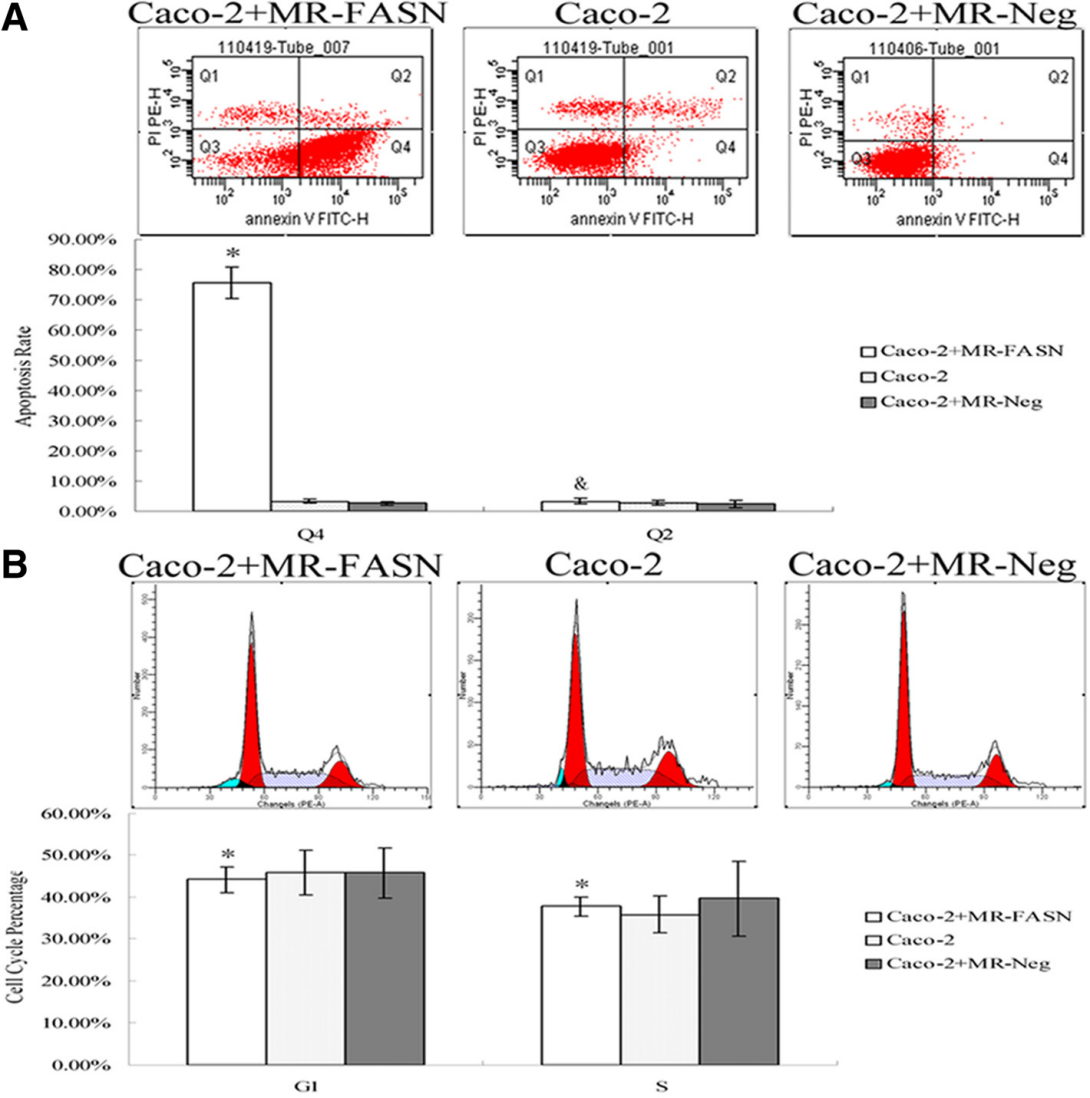
**The apoptosis and cell cycle of Caco-2 cells by RNA interference.** (**A**) The apoptosis rate. * Compared with two control groups, *P* < 0.05. ^&^ Compared with two control groups, *P* > 0.05. (**B**) The cell cycle percentage. * Compared with two control groups, *P* > 0.05.

Meanwhile, FASN silence led to a higher early apoptosis rate (75.57% ± 0.05) in Caco-2 cells (*P* < 0.05) (Figure 5A). Rather, there was no difference in the late apoptosis rate between FASN silenced and control cells (3.33% ± 0.01, 2.83% ± 0.01 and 2.53% ± 0.01) (*P* > 0.05) (Figure 5A). Additionally, no significant changes in cell cycle were observed upon FASN silence (*P* > 0.05) (Figure 5B).

## Discussion

Altered metabolism in human cancers has long been recognized. Endogenous fatty acid biogenesis catalyzed by the lipogenic enzymes such as FASN constitutes an oncogenic stimulus that drives the normal epithelial cells progression toward malignancy [17,18]. Intriguingly, recent experimental evidence supports the notion that the oncogenic nature of FASN-associated lipogenesis closely depends on the activity and expression of key cancer-related oncogenes such as HER2 [19,20]. HER2 overexpression leads to constitutive upregulation and maintenance of an exacerbated FASN-catalyzed endogenous fatty acid biogenesis [21,22], a “lipogenic benefit” in terms of enhanced cell proliferation, survival, chemoresistance and metastasis [14,23]. Conversely, disturbance of the lipogenic phenotype rapidly switches-off the oncogenic activity of the HER2 signaling platform, ultimately resulting in apoptotic tumor cell death [24,25]. Furthermore, the sole activation of endogenous fatty acid biosynthesis in non-cancerous epithelial cells is sufficient to induce a cancer-like phenotype functionally dependent on HER2 activity [20]. These findings reveal that HER2 oncogene establishes a positive bidirectional relationship with FASN, in this way strictly ensuring a hyperactive de novo fatty acid biogenesis.

In the current study, we demonstrated that RNAi-mediated inhibition of FASN dramatically reduced the expression of HER2, PI3K and Akt in colorectal cancer cells. It implies that FASN can effectively regulate the “HER2-PI3K/Akt axis” activity of colorectal cancer cells. Considering that HER2 overexpression stimulates the activity of FASN and ultimately mediates increased endogenous fatty acid biosynthesis [10,26], these findings implies a bidirectional connection between FASN and HER2 in colorectal cancer cells. Additionally, it also suggests that FASN is not only associated with various signaling pathways regulating proliferation, metabolism and survival in colorectal cancer cells, but also controls genes inducing malignant transformation in colorectal oncogenesis.

Recent evidences indicate that cancers with high expression of FASN always undergo a significant endogenous fatty acid biosynthesis and display a biologically aggressive subset [27,28]. Moreover, the upregulation of FASN expression is an early event in cancer development [29], it is more pronounced in advanced tumors, and correlates with a poor prognosis [27]. Importantly, we and others have demonstrated that inhibition of FASN with pharmacological inhibitors is selectively cytotoxic to human cancer cells and leads to a significant antitumor effect [30,31], suggesting that activation of fatty acid synthesis is required for carcinogenesis.

In this study, we observed that RNAi of FASN expression blocked the proliferation and migration of colorectal cancer cells and increased apoptosis rate. Furthermore, the decreased proliferation and migration of colorectal cancer cells could be partly attributed to the decreased activity of “HER2-PI3K/Akt axis” what was regulated by FASN. It implies that FASN plays a central role in the malignant phenotype maintenance of colorectal cancer cells by enhancing cancer cell survival and proliferation. All these findings suggest that FASN may be used for diagnosis, prognosis, early intervention, and treatment of various human cancers. However, further studies would be necessary to understand the role of FASN in the carcinogenesis.

As a large protein with a complex structure and multiple catalytic domains, FASN is considered as an important metabolic enzyme and a potential target in human cancers. Elevated FASN expression appears to be an early event in the tumorigenesis, and it is regulated by several signaling pathways. Elevated FASN expression in cancer cells seems to modulate lipid raft domains and various biological processes which in turn prevent apoptosis and promote cell survival. However, the detailed mechanism on how FASN regulates these biological processes is currently unknown. Although it is now known that FASN may be a proto-oncogene and its overexpression promotes tumorigenesis and survival, how FASN is upregulated in normal or pre-neoplastic cells to promote tumorigenesis is unclear, which requires and deserves further investigation.

## Competing interests

The authors declare that they have no competing interests.

## Authors’ contributions

NL wrote the manuscript, and also participated in the execution and analysis of this study with HL, CC and XB. PH participated in the design and analysis of this study. All authors read and approved the final manuscript.
